# Structural heterogeneity of the rat pulmonary vein myocardium: consequences on intracellular calcium dynamics and arrhythmogenic potential

**DOI:** 10.1038/s41598-018-21671-9

**Published:** 2018-02-19

**Authors:** C. Pasqualin, A. Yu, C. O. Malécot, F. Gannier, C. Cognard, D. Godin-Ribuot, J. Morand, P. Bredeloux, V. Maupoil

**Affiliations:** 1 CNRS ERL 7368, Signalisation et Transports Ioniques Membranaires, Equipe Transferts Ioniques et Rythmicité Cardiaque, Groupe Physiologie des Cellules Cardiaques et Vasculaires, Tours, France; 2 CNRS ERL 7368, Signalisation et Transports Ioniques Membranaires, Equipe Transferts Ioniques et Rythmicité Cardiaque, Poitiers, France; 3 Laboratoire Hypoxie PhysioPathologie, INSERM U1042 Grenoble, France

## Abstract

Mechanisms underlying ectopic activity in the pulmonary vein (PV) which triggers paroxysmal atrial fibrillation are unknown. Although several studies have suggested that calcium signalling might be involved in these arrhythmias, little is known about calcium cycling in PV cardiomyocytes (CM). We found that individual PV CM showed a wide range of transverse tubular incidence and organization, going from their virtual absence, as described in atrial CM, to well transversally organised tubular systems, like in ventricular CM. These different types of CM were found in groups scattered throughout the tissue. The variability of the tubular system was associated with cell to cell heterogeneity of calcium channel (Ca_v_1.2) localisation and, thereby, of Ca_v_1.2-Ryanodine receptor coupling. This was responsible for multiple forms of PV CM calcium transient. Spontaneous calcium sparks and waves were not only more abundant in PV CM than in LA CM but also associated with a higher depolarising current. In conclusion, compared with either the atrium or the ventricle, PV myocardium presents marked structural and functional heterogeneity.

## Introduction

Ectopic electrical activity originating from the pulmonary veins (PV) myocardial sleeves has been found to trigger and maintain paroxysmal atrial fibrillation in humans^1^. Although triggered activity, re-entry mechanisms and abnormal automaticity have been proposed, the exact mechanism initiating the rapid electrical firing of PV foci that propagate to the atria remains unknown^2–5^. Several observations^5–10^ have suggested that the calcium cycle of PV cardiomyocytes (CM) may be involved in the generation of abnormal electric signals which could trigger supraventricular arrhythmias. Moreover, in a model of catecholaminergic automatic activity in PV, repetitive bursts of slow type action potentials are not suppressed by the block of Na_v_1.5 channels, supporting their reliance on calcium^11,12^.

In the heart, calcium entering the cell through voltage-activated L-type calcium channels (Ca_v_1.2) localised on the sarcolemmal membrane and in transverse tubules (TT) binds to and activates type 2 ryanodine receptors (RyR2) which release the calcium stored in the sarcoplasmic reticulum (SR). This process of calcium-induced calcium release provokes an increase in intracellular calcium concentration known as the calcium transient. In ventricular CM, calcium channels localised on the well-developed and organised TT network are distributed throughout the entire cell and close to RyR2, thus provoking a homogeneous calcium release. In atrial myocytes, where the TT network is poorly developed and not transversally organised, calcium channels are mainly situated on the sarcolemmal membrane at the cell periphery. Calcium entering the cell has to diffuse to reach the RyR2 and thus provokes a non-homogeneous calcium release^13^. Therefore, the spatial organisation of Ca_v_1.2 and RyR2 has a major impact on the spatiotemporal shape of calcium transients, which can play a significant role in the cellular contractile properties and likely in arrhythmogenicity^13,14^.

A better understanding of calcium dynamics in PV CM could help to identify trigger mechanisms of atrial fibrillation. Therefore, the aim of this study was to explore the TT system, the localization of Ca_v_1.2 and RyR2 and calcium transients in the rat PV CM population. Since we found heterogeneities at both cellular and tissue levels in PV CM, comparisons with the left atria (LA) and the left ventricle (LV) were performed to determine differences which might explain the role of PV CM in the triggering of paroxysmal atrial fibrillation.

## Results

### Transverse tubular networks in isolated PV CM

**Figure 1:**
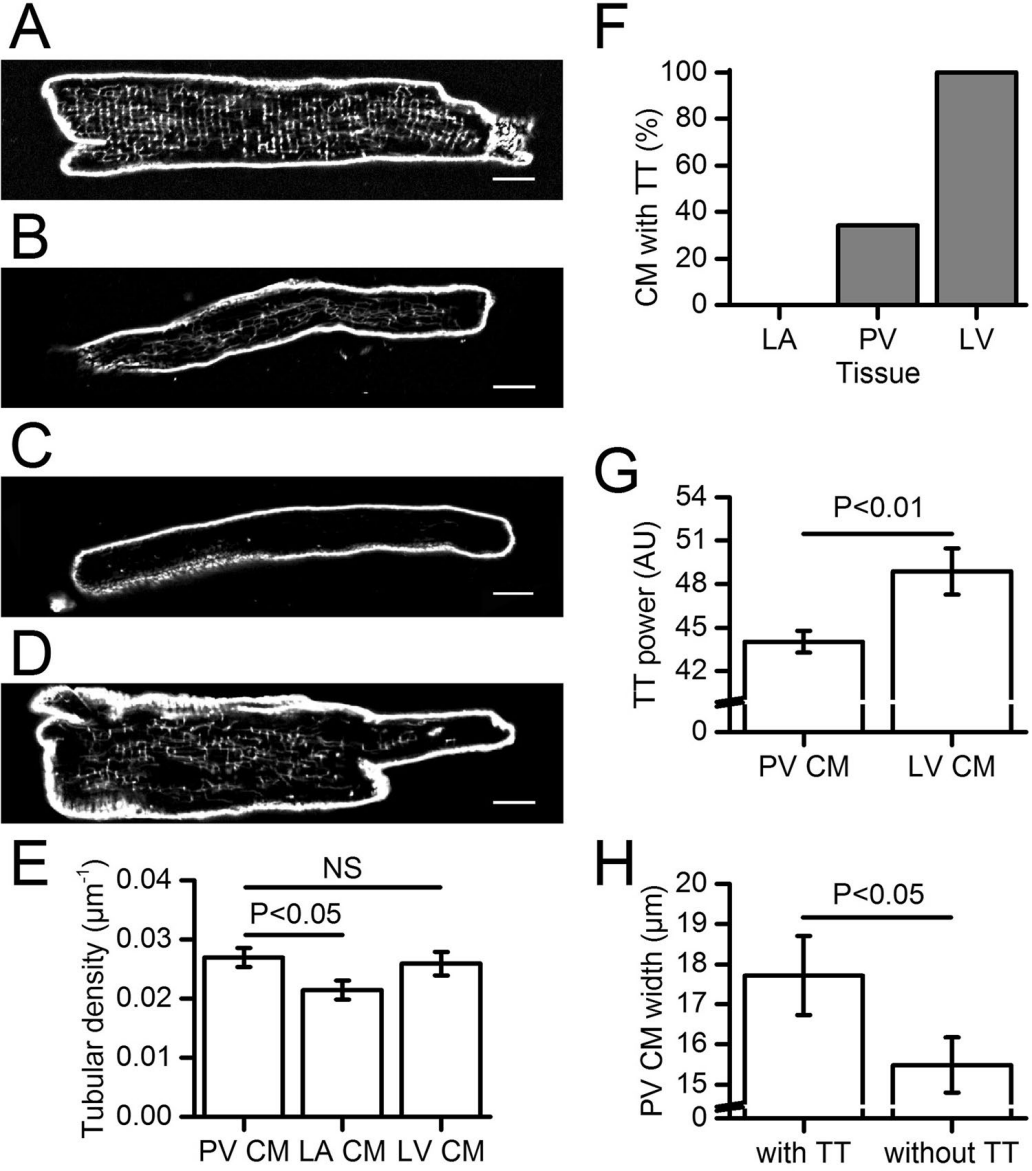
Confocal microscopy of di-8-ANEPPS labelled tubular networks in isolated PV, LA and LV CM. (**A**–**D**) represent examples of the different types of tubular networks found in the PV CM population. (**A**) CM with clearly organised TT, (**B**) CM with randomly distributed tubules, (**C**) CM without apparent tubular system and (**D**) CM with partially transversally organised tubular network. Scale bars represent 10 µm. (**E**) Tubular density in PV (n = 52), LA (n = 55) and LV CM (n = 15). (**F**) Percentage of CM with visually apparent and clearly organized transverse TT network in LA (n = 55), PV (n = 82) and LV CM (n = 15). (**G**) Transverse regularity of the TT network (TT power) in those PV with TT (n = 28) and LV CM (n = 15). (**H**) Width of PV CM with (n = 28) and without TT (n = 32).

Membrane staining with di-8-ANEPPS revealed wide variation in membrane invaginations in the isolated PV CM selected under light transmission (Fig. 1). This ranged from CM displaying a highly developed tubular network with a regular transverse organisation (Fig. 1A), as described for LV CM, to CM with sparse and disrupted network (Fig. 1B) or without tubule (Fig. 1C) as observed in LA CM. Moreover, some PV CM showed heterogeneous tubular organisation consisting of transversally organised tubules in one part of the cell and no tubule in the other (Fig. 1D). When all PV CM data were combined, the density of the tubular network was similar in PV (n = 52) and LV CM (n = 15), but greater in PV than in LA CM (n = 55) (Fig. 1E).

The analysis of the tubular network organisation showed that among PV CM, 34% (n = 28/82, n_rats_ = 4) presented a regular transverse labelling similar to the transverse tubules (TT) observed in 100% of LV CM (Fig. 1F). TT spacing regularity, quantified by the TT power, was significantly less in PV CM (43.4 ± 0.3, n = 28) than in LV CM (48.5 ± 1.7, n = 15; P < 0.01; Fig. 1G). Moreover, the tubular density in these PV CM (0.033 ± 0.002 µm^−1^, n = 28) was significantly higher than in LV CM (0.026 ± 0.002 µm^−1^, n = 15, P < 0.05). In these PV CM, there was no correlation between the tubular density and the transverse organisation level.

The remaining 66% (n = 54/82) of PV CM presented a tubular network comparable to that of LA CM *ie* ranging from sparse and irregular networks to no internal tubules at all. Overall, the PV CM tubular density (0.023 ± 0.002 µm^−1^, n = 32) was similar to that of LA CM (0.021 ± 0.002 µm^−1^, n = 55, n_rats_ = 3) and it was also positively correlated to the cell width (Fig. S1 in the supplementary data) as already described in LA CM by Smyrnias *et al*.^15^.

To distinguish these two PV CM populations in the remaining part of our study, the term “transverse tubules” (TT) has been restricted to tubular networks presenting a detectable transversal organisation.

PV CM with TT were significantly wider than those without TT (Fig. 1H). However, PV CM with TT were narrower than those of the LV (17.7 ± 1.0 µm, n = 28 *vs*.21.5 ± 1.5 µm, n = 15; P = 0.02) whereas cell widths were similar in PV CM without TT and LA CM (15.5 ± 0.7 µm, n = 32 *vs*. 16.0 ± 0.6 µm, n = 55; P = 0.68).

Because of the heterogeneity of isolated PV CM regarding their tubular network organisation (i.e., tubules with or without transverse organisation, no or poorly developed tubules) we investigated their incidence and spatial distribution within the pulmonary veins sleeves.

### Distribution of CM with different types of tubular networks within PV myocardial sleeves

**Figure 2:**
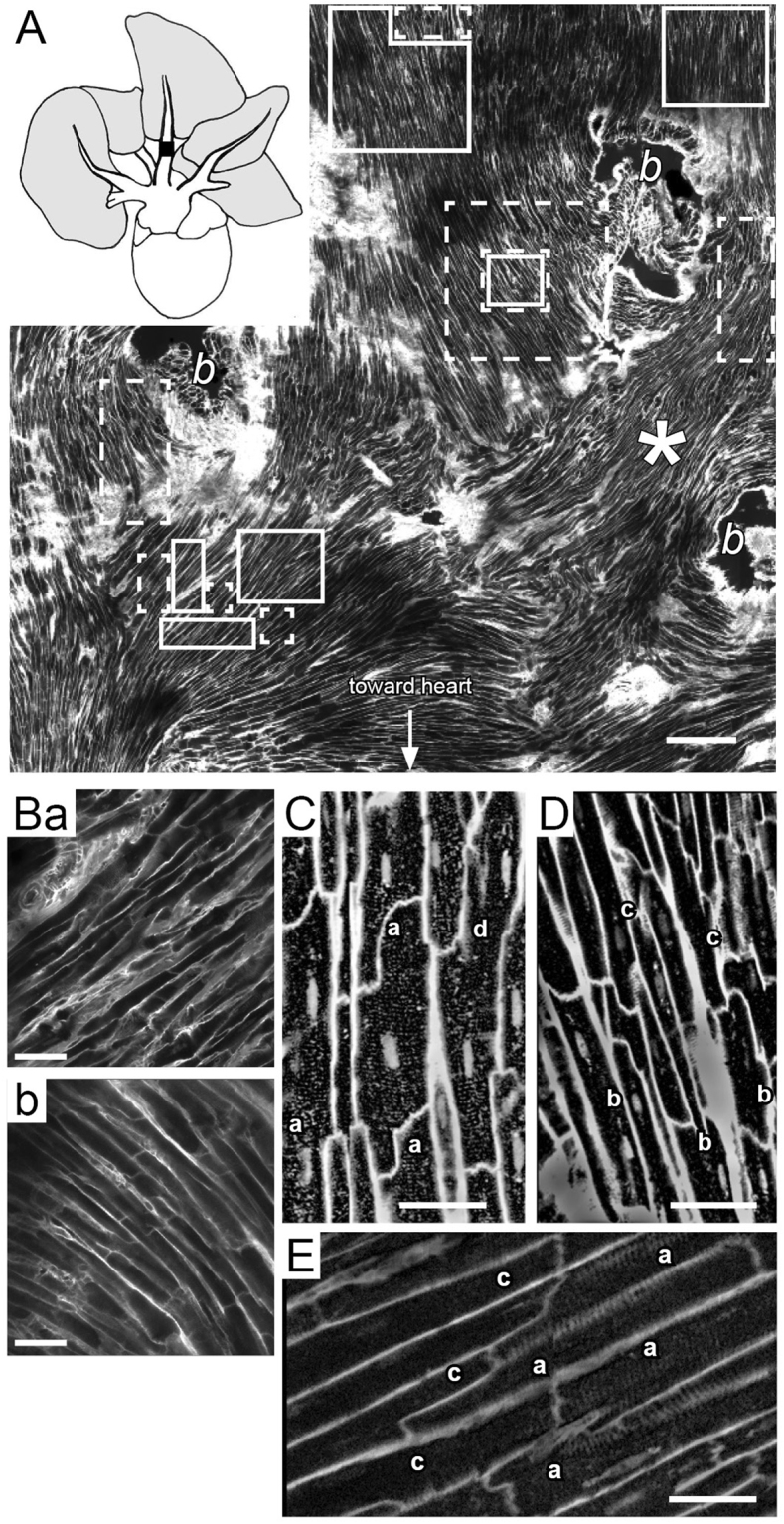
Myofibrils orientation and distribution of the different types of CM in whole-mounts of PV stained with wheat germ agglutinin (WGA) and observed in confocal microscopy. (**A**) Complex orientations of CM on the luminal surface of one of the right superior PV (inset). Scale bar represents 200 µm. ‘*b*’ indicate ostia of small veins bifurcating from the main. (**B**) Orthogonal arrangement of consecutive layers of CM in the PV in the zone indicated by the star in A. (a) Was recorded on the luminal surface and (b) 25 µm deeper into the tissue towards the abluminal surface. (**C**) Example of CM presenting a transverse tubular organisation, found in the areas delimited by the solid line boxes in A. (**D**) Example of CM with no TT found in the areas delimited by the dashed line boxes in A. (**E**) Example of an abrupt transition between CM with and without TT. The four patterns of tubular system found in isolated PV CM (Fig. 1) are also seen in the intact pulmonary vein as shown in panels C to E: (a) CM with transverse tubules; (b) CM with randomly distributed tubules; (c) CM without or with low extended tubular system; and (d) CM with partially transversally organised tubular system. Scale bars represent 30 µm. The results shown in this figure are representative of those found in 7 rats.

Cardiomyocyte membrane labelling with WGA in the whole PV myocardial sleeves showed complex and variable orientations of PV CM (Figs 2A and S2A) especially near the ostia of secondary PV branching from the main (Fig. 2A). Furthermore, consecutive CM layers were often almost orthogonally orientated (Fig. 2Ba,Bb) whereas a unidirectional orientation of consecutive cardiomyocyte layers was observed in LA.

PV CM with different tubular networks found in isolated cells (Fig. 1) were also observed in WGA-labelled whole mounts of PV tissue. They were found throughout the muscle sleeves in both luminal and abluminal layers (Fig. 2). Individual CM with a given tubular pattern were not scattered through the vein but grouped together (Fig. 2A,C–E). These groups varied in size from tens of cardiomyocytes to several square millimetres in area. Figure 2A illustrates the position and approximate size of some of these groups (boxes) in one vein. It also shows areas where PV CM with one type of tubular network were surrounded by PV CM with another type of network (boxes within boxes). The distribution of these groups showed no recognisable pattern or association with anatomical features either within or between different whole mount preparations of the pulmonary vein (n = 7, two other examples are shown in Fig. S3). In particular, there was not a proximal to distal gradient of cell type along the vein and there was no evidence of an abrupt change in tubular network type at the junction between PV and the roof of the LA. On the other hand, CM of different tubular network type could be directly connected (Fig. 2E).

These observations in PV sleeves sharply contrasted with those in the LA where the CM were more uniform regarding their tubular network organisation (Fig. S4).

### RyR2-Ca_v_1.2 spatial distribution in PV CM vs.LA and LV CM

To examine the consequence of the variability of tubular organisation on the calcium transient, the distribution of RyR2 and Ca_v_1.2 were compared in PV, LA and LV CM.

**Figure 3:**
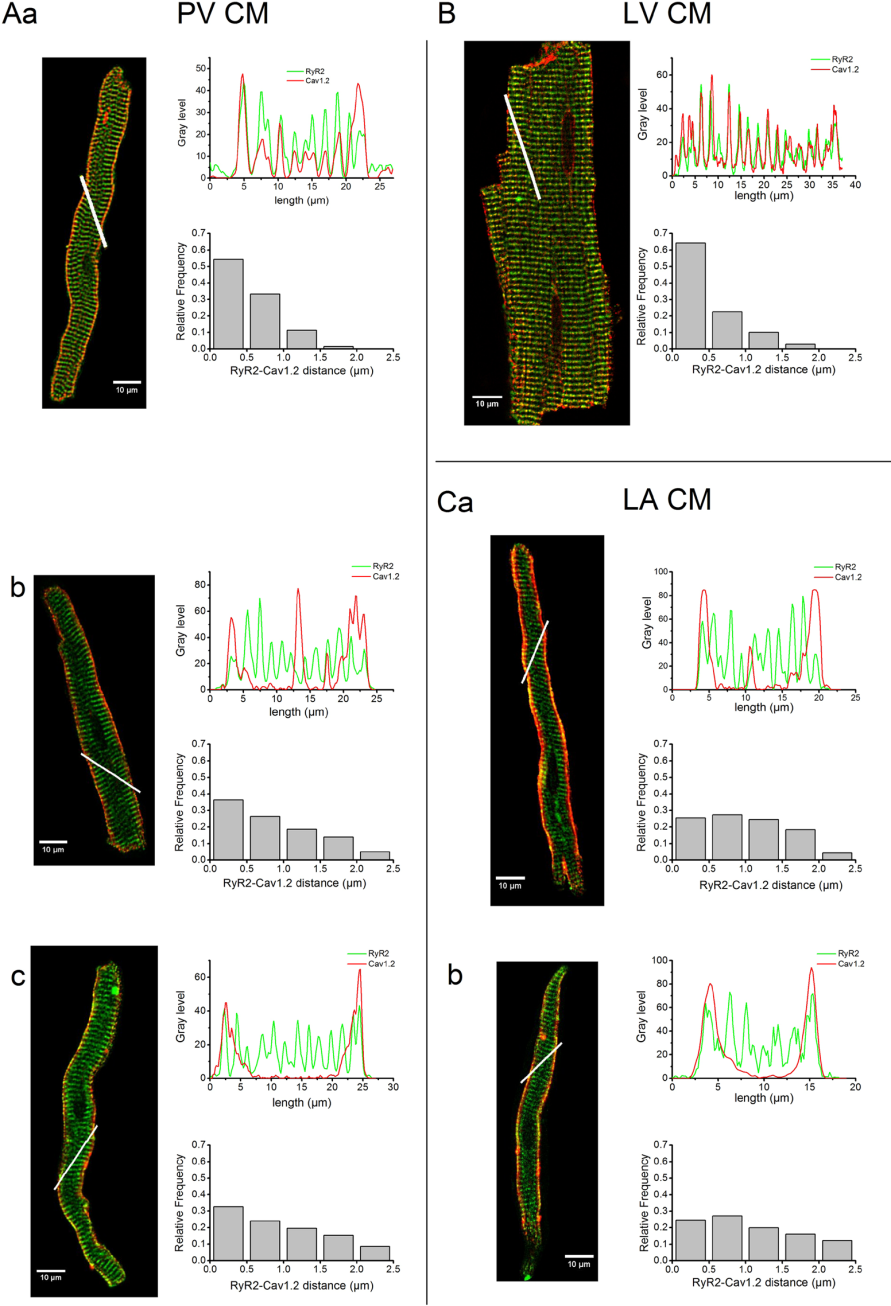
Representative images of isolated CM of PV (**A**), LV (**B**) and LA (**C**) with fluorescent RyR2 (green) and Ca_v_1.2 (red) double immunostaining recorded with confocal microscopy. For each cell, RyR2 and Ca_v_1.2 fluorescence intensity profiles along the white line were traced in a graph and the histograms represent the distribution of the distances between each RyR2 cluster and its nearest Ca_v_1.2 cluster calculated across the entire cell slice. Aa and B illustrate a high level of RyR2-Ca_v_1.2 co-localisation. Ab and Ca illustrate CM where RyR2 and Ca_v_1.2 are only partially co-localised. Ac and Cb show CM where RyR2 and Ca_v_1.2 are co-localised only at the sarcolemmal membrane.

RyR2 were present in the entire cell volume in each of PV, LA and LV CM (Fig. 3). This formed a transverse striated pattern with spacing identical to that of the sarcomere (Fig. S5) (n > 100 CM for each tissue). Therefore, irrespective of t-tubule presence or organization, all CM possessed a full and organized RyR2 distribution.

In contrast, Ca_v_1.2 distribution in PV CM varied from cell to cell (n = 36). Some PV CM displayed a well striated pattern of Ca_v_1.2 labelling (Fig. 3Aa) as in LV CM (Fig. 3B) whereas some others were only labelled at the cell periphery (Fig. 3Ac) as in LA CM (Fig. 3Cb). A variety of intermediate distribution profiles were also observed in some PV and LA CM where the exact position, number and grey scale amplitude varied (1 each in Fig. 3Ab,Ca). Moreover, the three distribution profiles were also simultaneously present to various extents in some of the PV CM observed. These findings were consistent with the study of the tubular network (Fig. 1) since Ca_v_1.2 distribution seemed to reflect the extent and organisation of tubules within the CM.

Co-localisation of RyR2 and Ca_v_1.2 varied greatly among PV CM (n > 25 and n_rats_ ≥ 2 for double labelling of RyR2-Ca_v_1.2 in each tissue). Superimposition of the grey level profile plots of the double immunostained CM showed that RyR2-Ca_v_1.2 co-localisation was either present across the entire cell as in LV CM (Fig. 3Aa,B) or only at the cell periphery (Fig. 3Ac,Cb) or irregularly along the line scan (Fig. 3Ab,Ca). This was confirmed by the analysis of the distribution frequency of the RyR2-Ca_v_1.2 distances showing a higher frequency for the shortest distances for some PV CM and the LV CM (Fig. 3Aa,B) and more equally distributed distances for other PV CM types and LA CM (Fig. 3Ab,c,Ca,b).

Thus, the spatial proximity of RyR2 and Ca_v_1.2 was heterogeneous in PV CM and could therefore lead to different intracellular calcium handling. The intracellular calcium transients were then studied in stimulated PV CM.

### Spatiotemporal shapes of electrically evoked calcium transients in PV CM

**Figure 4:**
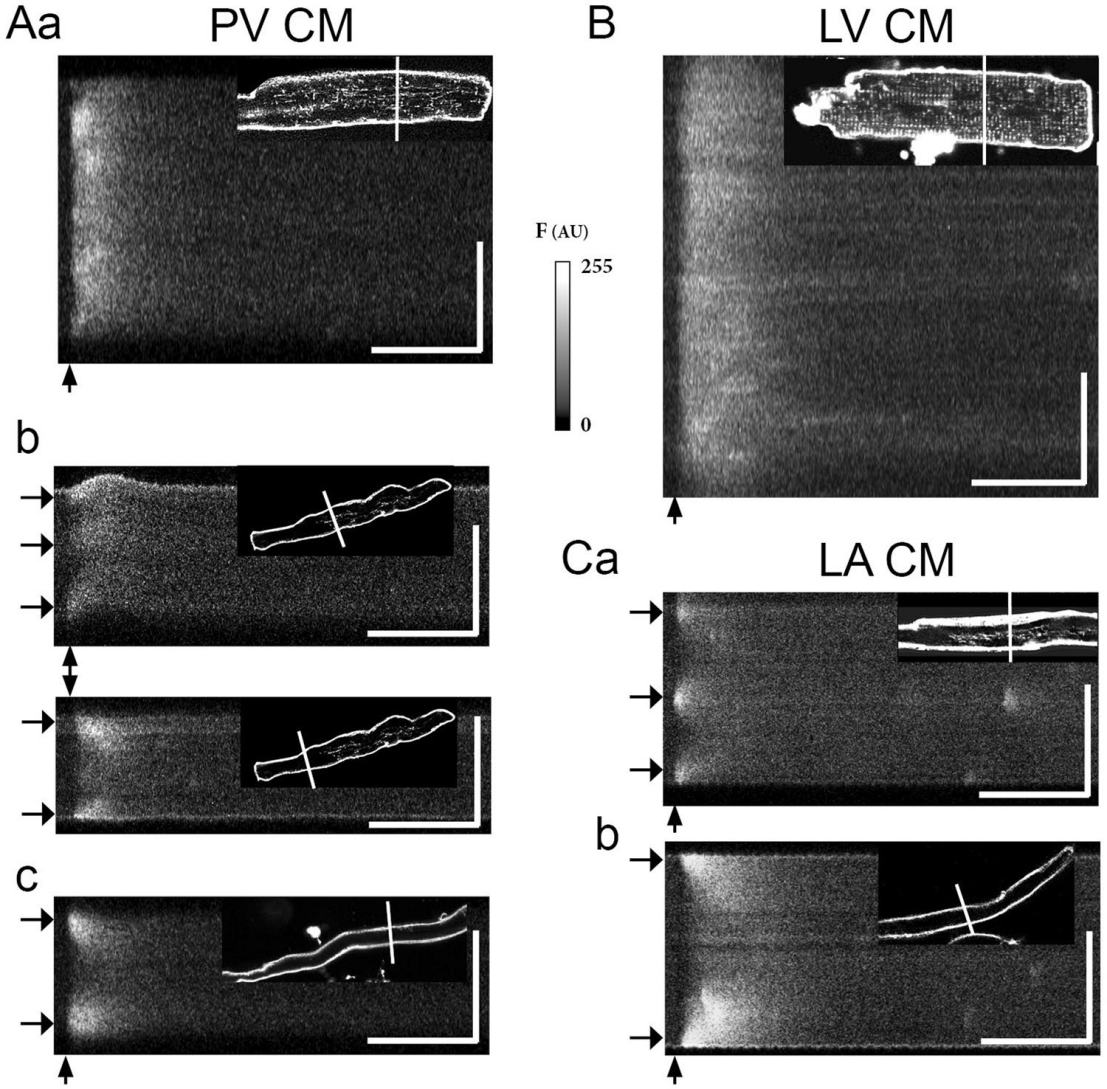
Examples of calcium transients recorded upon electrical stimulation of isolated PV (**A**), LV (**B**) and LA (**C**) CM. Different forms are observed in confocal line scan images of Fluo-4 AM stained CM recorded transversally to their longitudinal axis (white lines in the insets). Three shapes of Ca^2+^ transient are illustrated: *I*-shaped (Aa and B), *W*-shaped (Ab upper panel and Ca) and *U*-shaped (Ab lower panel, Ac and Cb). Horizontal arrows indicate the Y-axis (space) position of initial calcium increase for *W*- and *U*-shaped Ca^2+^ transients. Vertical arrows indicate the onset of the electrical field stimulation pulse. Vertical and horizontal scale bars represent respectively 10 µm and 200 ms. n > 25 CM for each tissue. For each linescan, the corresponding CM image with di-8-ANEPPS labelled tubular network is inset. It should be noted that the two panels shown in Ab were recorded from the same CM.

As expected, the Ca^2+^ transient in response to field stimulation observed on linescan XT images oriented transversally to the cell longitudinal axis had different shapes in PV CM depending on their tubular network pattern (insets in Fig. 4). PV CM presenting well-developed regular transverse tubules showed *I*-shaped Ca^2+^ transient like LV CM (Fig. 4Aa,B), *i*.*e*., the calcium release was almost homogeneous across the cell. On the other hand, in cells exhibiting various degrees of tubular density and organisation, the rise of cytoplasmic calcium concentration was asynchronous along the scanned line. In PV and LA CM showing irregular tubules, *W-*shaped Ca^2+^ transients, *i*.*e*., originating from different points of the cell, could be observed (Fig. 4Ab,Ca). In PV CM with little or no tubular network, the Ca^2+^ transient started close to the sarcolemma and spread towards the cell centre, giving rise to a *U*-shaped transient as observed in some of the LA CM (Fig. 4Ac,Cb). Finally, different Ca^2+^ transients could be recorded in the same PV CM (Fig. 4Ab) when tubules were restricted to only part of the CM (Fig. 1D). In PV CM, the amplitudes (F/F_o_) of *I*- and *W*-shaped Ca^2+^ transients were larger than those showing *U*-shaped transients (Fig. S6).

**Figure 5:**
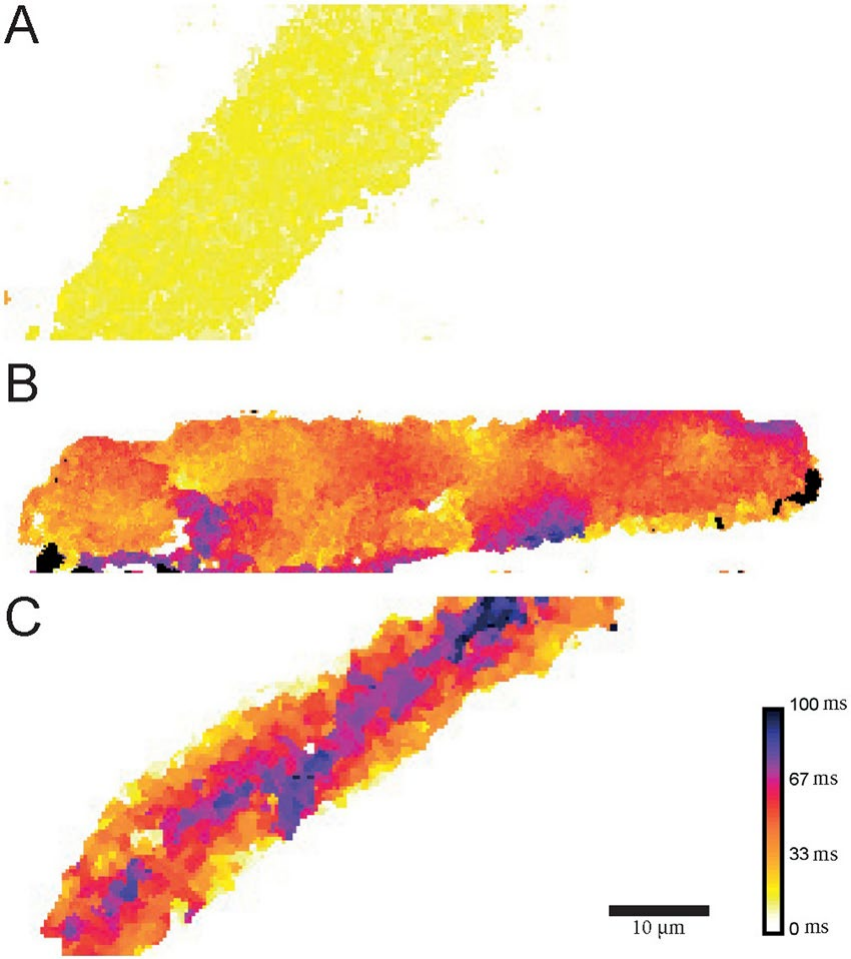
Examples of calcium increase delay maps of calcium transients with distinct spatiotemporal shapes. These were recorded in Fluo-4 AM loaded PV CM with confocal microscopy. Pseudo colours indicate the delay between the start of the electrical field stimulation and the time of the maximum rate of rise of calcium in the cytoplasm. (**A**) A homogeneous and rapid increase of calcium in the whole CM following stimulation. (**B**) Following stimulation calcium rise occurs rapidly in some parts of the CM and later in others. (**C**) Calcium rises first near the plasma membrane at the periphery of the CM. Then it propagates centripetally to the centre of the CM. Calcium transients from n = 20 PV CM were analysed.

High frequency XYT laser scanning images of PV CM planes were also recorded to give a more complete view of calcium transient spatiotemporal shapes in the whole CM. Such calcium transients recorded on a central PV CM plane are shown as “calcium increase delay maps” in Fig. 5. Spatiotemporally homogeneous (uniform pseudo colour; Fig. 5A) and heterogeneous (multiple pseudo colours; Fig. 5B,C) calcium transients were observed. For *W*-shaped calcium transients (Fig. 5B), calcium rise occurred early at several locations within the CM including its centre. Outside these locations, the calcium rise occurred with a delay increasing with the distance from the initial calcium release site. On the other hand, for *U*-shaped calcium transients (Fig. 5C), calcium rise occurred firstly near the plasma membrane at the CM periphery, and then propagates toward the centre of the CM. The calcium transients corresponding to each calcium increase delay map presented in Fig. 5 can be visualised in their respective videos (slowed down 20 times for illustrative purpose) in the supplementary data.

This first part of our study showed that the structural heterogeneity of PV CM with regards to their tubular network has functional consequences for calcium release in stimulated CM. We focus the second part of our study on spontaneous calcium events in PV and LA CM.

### Spontaneous calcium sparks in PV CM and LA CM

**Figure 6:**
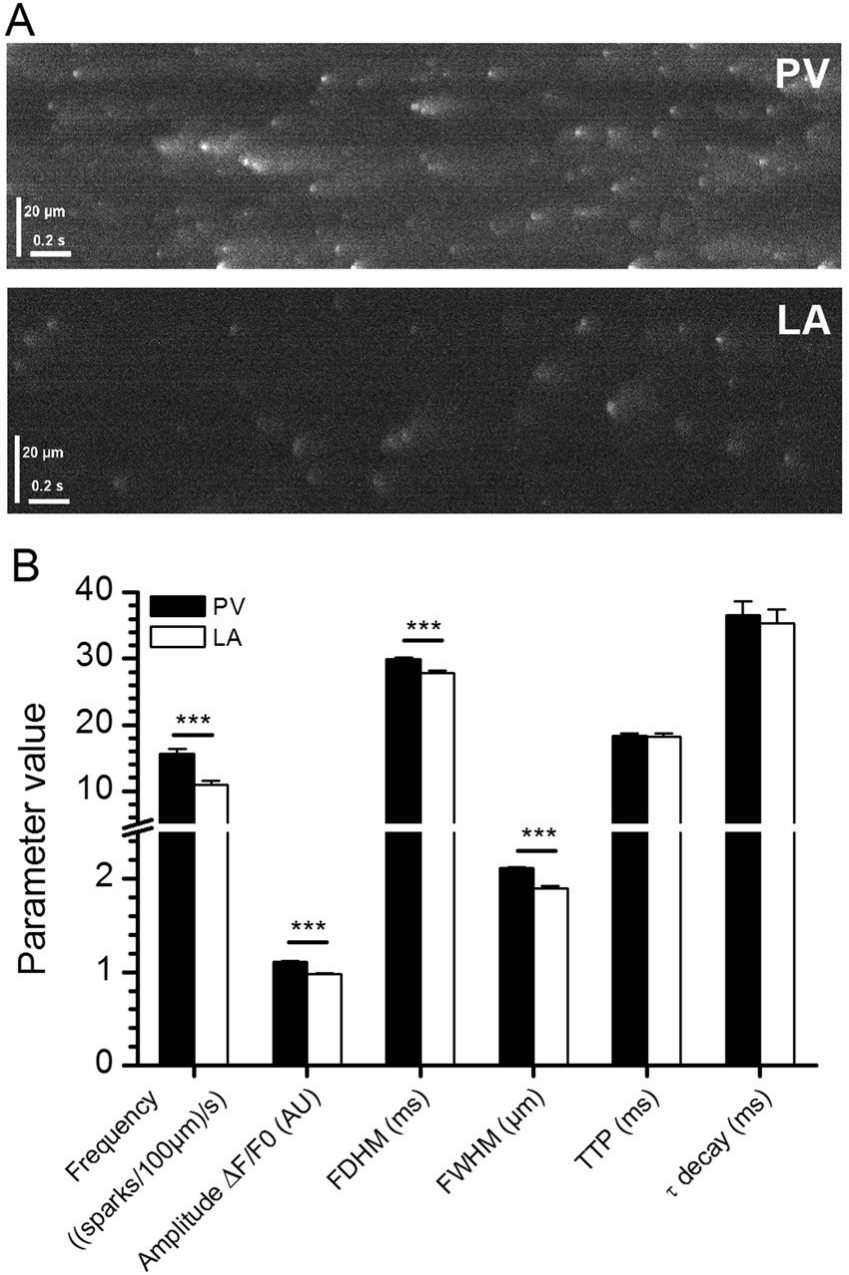
Spontaneous calcium release events in rat PV and LA CM and their characteristics at 32 °C. (**A**) Representative traces of spontaneous sparks (Fluo-4 AM) observed by confocal microscopy in PV (upper trace) and LA (lower trace) CM. (**B**) Spontaneous spark characteristics. FDHM: Full Duration at Half Maximum; FWHM: Full Width at Half Maximum; TTP: Time to Peak. Data are mean values ± SE of 6003 and 3182 sparks analysed in 132 PV (4 rats) and 120 LA (4 rats) cardiomyocytes, respectively. ***P < 0.001 *vs*. PV (ANOVA followed by Holm-Sidak’s posthoc test).

Spontaneous calcium sparks, also called spontaneous calcium events (SCaEs), occur when RyR calcium release channels spontaneously open at diastolic calcium concentration^16,17^. From the representative recordings of SCaEs shown in Fig. 6A, it is clear that sparks occur more frequently in PV CM than in LA CM (Fig. 6B). Their amplitude, duration and width were also higher in PV than in LA (Fig. 6B). The spark times to peak and decay time constants were identical in PV and LA CM.

### SR calcium load in PV and LA CM

Since spontaneous sparks are highly dependent on the SR calcium load^17^, the latter was estimated from the integral of the sodium-calcium exchange (NCX) current in response to a rapid application of 10 mM caffeine. The results are presented in Table S1. In our experimental conditions, the absolute SR calcium amount that could be extruded via the NCX was not significantly different in PV (2.32 ± 0.16 fmol, n = 21) and LA CM (2.23 ± 0.19 fmol, n = 15). Taking the capacitance to volume ratio of 14.6 pF/pL determined in rat left atria^18^ and our experimental values of cell capacitance, the SR calcium concentration relative to the total cell volume amounted to ~600 µM in LA CM. To our knowledge, the capacitance to volume ratio has not been determined for rat PV CM. Also, such a mean ratio is not appropriate for PV CM because of the large variability of the amount of tubular network which contributes to the cell capacitance. Thus, considering that PV CM share characteristics of both LA and LV CM with regards to their tubular network and size, we have assumed that the extreme values of PV CM capacitance to volume ratio were those of LA (14.6 pF/pL) and LV CM (6.76 pF/pL^19^). Taking these values, SR calcium concentration could be estimated to ~180–380 µM of total cell volume in PV CM, i.e., less than in LA CM.

### Calcium waves and corresponding inward current

The high frequency of spontaneous calcium sparks in PV CM might be determinant for triggering arrhythmias: a large number of simultaneously occurring sparks in a limited cytoplasmic volume can initiate propagated waves of calcium-induced calcium release. These waves have been shown to induce delayed afterdepolarisations (DAD) in heart failure^20^ and atrial fibrillation^21,22^. Thus, wave frequency and their corresponding induced depolarising current might play a key role in the generation of ectopic foci in PV which occur during atrial fibrillation.

Calcium wave frequency determined in intact CM was significantly higher in PV CM (5.5 ± 1.6 waves/90 s, n = 40) than in LA CM (0.8 ± 0.4 waves/90 s, n = 22; P < 0.05).

**Figure 7:**
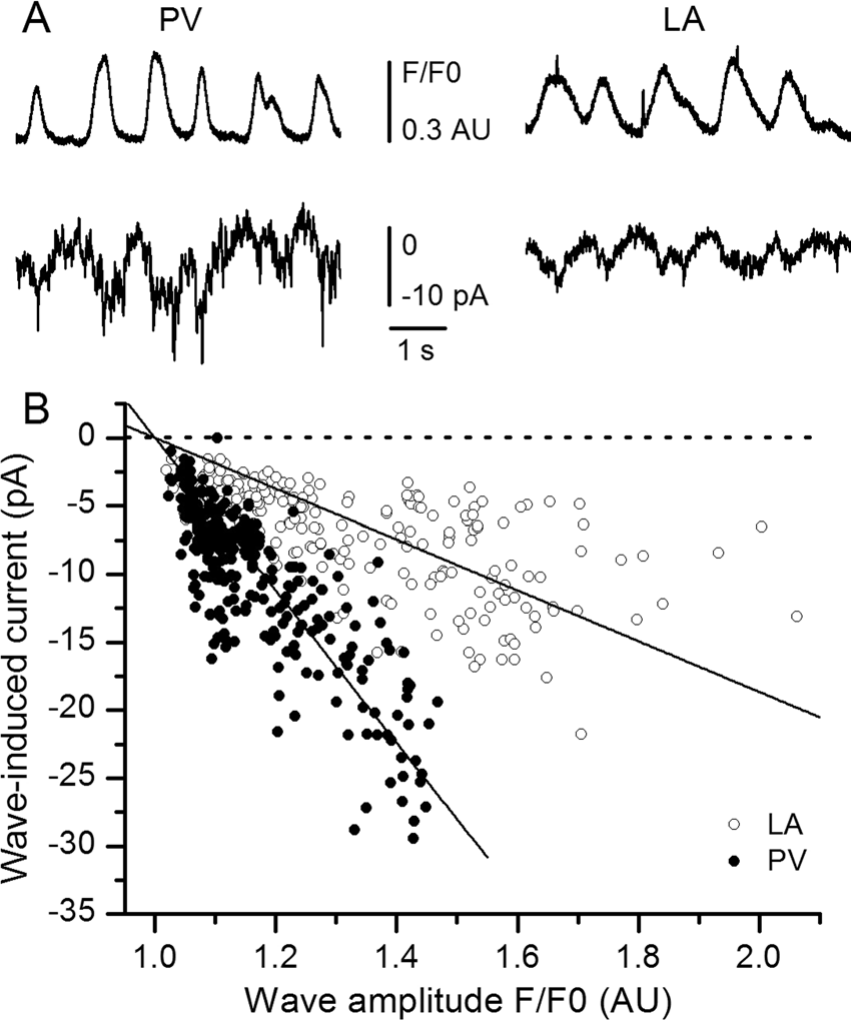
Spontaneous calcium waves and induced currents in PV and LA CM. (**A**) Examples of calcium wave fluorescence (top traces) and simultaneous variations of the holding current at −70 mV (lower traces) in PV and LA CM. Downward deflections correspond to inward currents. **(B**) Inward current amplitude induced by spontaneous calcium waves occurring at rest at −70 mV. Each data point corresponds to a wave recorded in LA (open symbols, n = 242 waves, 9 CM) or in PV CM (filled symbols, n = 272 waves, 9 CM). Straight lines represent linear adjustments of the form Y = AX + B to the data points with the constraint current = 0 for F/F0 = 1. Slopes for PV and LA are statistically different (P < 0.001; Student’s *t*-test).

Spontaneous calcium waves and their corresponding currents were therefore studied in whole-cell patch-clamp experiments. Considering their low frequency, we used a stimulation protocol (see supplementary data) aimed at increasing intracellular calcium concentration and allowing full SR calcium replenishment. In these conditions, calcium waves frequency was significantly increased (to 48.8 ± 7.7 waves/90 s in PV CM, n = 9, and to 40.2 ± 7.3 waves/90 s in LA CM, n = 9), thus facilitating their study. Figure 7A shows typical recordings of calcium waves fluorescence and concomitant variations of the holding current at −70 mV (a potential close to the resting potential), in PV and LA CM. Each calcium wave was associated with an inward current. It also seemed that for a similar amplitude, calcium waves appeared to be associated with larger currents in PV than in LA CM. Figure 7B illustrates the linear relationships (P < 0.001) existing between the amplitudes of the calcium waves and of the corresponding inward currents recorded at −70 mV in both PV and LA CM. Slopes of the linear regressions are statistically different (PV: −56.0 pA/AU; LA: −18.7 pA/AU; P < 0.001), indicating that inward currents generated by calcium waves are indeed larger in PV than in LA CM. This was also true when the current amplitudes were normalised to the cell capacitance, although the difference between the slopes was less pronounced (P < 0.05). Therefore, the arrhythmogenic potential of calcium waves appears more important in PV than in LA CM.

## Discussion

In the present study we show, for the first time, a structural and functional heterogeneity of PV CM associated with the organisation of the tubular system. These different types of CM were gathered in groups which seem randomly distributed in the PV muscle sleeves. Also, PV CM showed higher calcium spark and wave frequencies than LA CM. Waves which in PV CM generate a greater inward current than in LA CM.

Although PV myocardial sleeves are in continuity with the left atria, the tubular system of PV CM differs from that of LA CM. Historically LA CM were not considered to show an internal TT system^23,24^. Though more recently an organized transverse tubular network has been described in some LA CM^25,26^. Given the chosen analysis threshold beyond which we considered that tubules were transversally organized, we found LA CM either lacking an internal tubule system or having a sparse or disorganised network, but no LA CM with an organised one. Using the same criteria, we found one third of PV CM with a transversally organized tubular system similar to that of LV CM as well as cells with sparse and irregular networks and cells with no internal tubule systems. The presence in rat PV of CM with a transverse tubular organization was not surprising *per se* as this has been previously reported in tissue electron microscopy studies^27,28^ and di-8-ANEPPS staining of isolated CM^29^. Though Okamoto *et al*.^29^ did suggest that PV CM may be structurally heterogeneous and that they analysed only a subset of cells. To prevent bias in this study, cardiomyocytes were chosen under light transmission microscopy with appearance criteria (clearly visible sarcomeres and refractive membrane) to avoid selection under fluorescence microscopy. Moreover, an automatic analysis was performed with TTorg software^30^ in order to avoid operator bias. Therefore, the heterogeneity of PV CM observed in isolated cells is unlikely to be due to experimental artefacts. This is confirmed by the observation of these different types of CM in intact tissue stained with either di-8-ANEPPS or fluorescent WGA.

Since Ca_v_1.2 ion channels are localised on the sarcolemmal as well as tubule membranes Ca_v_1.2 distribution in PV cells ranged from a transversal organisation as in LV CM to a peripheral one as in most LA CM with all intermediate pictures reflecting the various tubular organisation levels. Consequently, various degrees of RyR2-Ca_v_1.2 coupling were observed among PV CM since RyR2 were always transversally organised.

These structural heterogeneities have functional consequences in terms of calcium handling by individual CM. Several calcium transient patterns which depend on the tubular organisation were observed in PV CM confocal line scan images: synchronous cytoplasmic calcium increase in the whole cell as in LV CM (highlighted by the classical *I* type of transient) and asynchronous cytoplasmic calcium increase (highlighted by the *U* and *W* types of transient) as depicted in LA CM^23,24^. Moreover, depending on the position of the line scanned on the CM image, *U* and *W* types of transient could be observed in a single PV CM. This heterogeneity of calcium transient shapes has been confirmed by XYT recordings which in addition provide an overview of calcium transient shapes in an entire CM plane.

Individual PV CM with or without transversally organised tubules are not randomly spread throughout the PV sleeves. They are gathered in groups which themselves appear to be irregularly distributed in the whole tissue. This patchwork of cell types in the PV may contribute to its arrhythmogenic potential. Thereby, the cluster organisation of CM together with the functional consequences for intracellular Ca^2+^ handling might lead to non-uniformity of excitation-contraction coupling within the PV myocardial sleeves. This could in turn lead more easily to the generation of arrhythmogenic calcium waves able to propagate within the PV. Such phenomenon were observed in a rat cardiac muscle model which mimicked the non-uniformity observed during a localized mechanical or ischemic damage of the myocardium^31^.

The cellular and tissue heterogeneities may also have functional consequences in terms of electric properties. Indeed, tubules are a key site for the regulation of action potential duration since de-tubulated rat ventricular CM have shorter action potential durations due to a reduced calcium influx^32^. Thus, if the presence and organisation of the tubular network also modulates PV CM action potential duration, localized discordance of APD, and consequent refractory period could facilitate the existence of ectopic foci, mostly due to re-entry phenomena. In addition, re-entry might be reinforced by the anisotropy of conduction caused by the complex myocardial fibres orientations observed in this study and by others in rat PV^33^ as well as in other species such as dog^34,35^ and humans^36^.

The higher frequency, amplitude, duration and width of calcium sparks in PV CM compared to LA CM is not related to a higher SR calcium load. But, it has been proposed that a close association between calcium channel and RyR2 are necessary for the initiation of spontaneous Ca sparks^24,37,38^ which then initiate propagated waves of calcium induced-calcium release^39^ in ventricular CM. Thereby, sparks might be more abundant in PV CM because of the better RyR2-Ca_v_1.2 coupling compared to LA CM. Indeed, in PV, 33% of CM present a transversally organised tubular network in close apposition to the sarcoplasmic reticulum.

Also the higher frequency and dimensions of calcium sparks in PV CM could be associated with higher frequency of calcium waves in intact CM. Moreover, for an equal relative increase in cytoplasmic calcium concentration during a wave, the inward current generated in PV CM is higher than in LA CM. Whatever the underlying mechanism which remains to be determined (*e*.*g*., larger NCX current and/or Ca^2+^-activated chloride conductance), this suggests that spontaneous calcium waves in PV CM are able to induce a larger depolarisation and therefore are more likely to trigger an arrhythmogenic action potential. This would be particularly true in pathological conditions such as Ca-overload. This hypothesis is strengthened by a recent observation in mice showing that spontaneous calcium release in PV CM could prevent their excitation by the sinus rhythm^8^ and created points of autonomous arrhythmogenic activity.

In conclusion this study reveals marked heterogeneity of structure and consequent function in myocytes within the cardiac muscle sleeves in pulmonary veins of the rat. This is in contrast with the relative homogeneity of CM in atrial and ventricular muscle. It remains to be determined whether these results translate to the PV of other species including human.

## Materials and Methods

### Cardiomyocyte isolation

All experiments involving animals were approved by the local institutional ethical committee (Comité d’Ethique en Expérimentation Animale Val de Loire) and carried out in accordance with European guidelines on animal experimentation and the French “Ministère de l’agriculture, de l’agroalimentaire et de la forêt”. LV, PV and LA cardiac myocytes were enzymatically isolated from 3–4 months old male Wistar rats as previously described^12^ and summarised in the supplementary data.

### Confocal observations on living cells

Cardiomyocytes were chosen based on their appearance in transmission microscopy to avoid selection bias: only myocytes with clearly visible sarcomeres and refractive membrane were used for these studies.

#### Tubular network and calcium transient

When studied alone, the tubular network of isolated cardiomyocytes was visualised in CM stained with 10 µM di-8-ANEPPS (Molecular Probes) with a confocal microscope (Olympus FV1000) equipped with an x60 oil immersion objective. Dye was excited at 488 nm and fluorescence collected between 505 and 605 nm.

For simultaneous calcium transients and tubular network studies, Fluo-4 AM (5 µM) and di-8-ANEPPS (Molecular Probes) double-labelled cells were used. To record calcium transients, CM were scanned with confocal microscopy (Olympus FV1000) at 500 Hz along a line perpendicular to their longitudinal axis avoiding the nuclear area. Fluo-4 and di-8-ANEPPS dyes were both excited at 488 nm and fluorescence intensities were collected between 505 nm and 525 nm and beyond 660 nm, respectively. During the acquisition, CM were electrically field stimulated with square pulses (1 Hz, 2 ms) and superfused at 32 °C with Tyrode’s solution. To record 2D calcium transients (XYT), the same experimental conditions were used except that the confocal microscope was a Dynascope Zeiss LSM710 NLO. Each video of calcium transients was acquired with a frame rate between 550 and 270 Hz depending on the cell size and orientation. Frame sizes were between 512 × 100 pixels and 512 × 200 pixels. Pixel size was 310 nm.

#### Calcium sparks

To record spontaneous calcium sparks, Fluo-4 AM loaded myocytes were superfused with Tyrode’s solution at 32 °C and observed on a confocal microscope (Olympus FV1000). Cells were line-scanned (XT) at 500 Hz along a longitudinal nuclear-free line with excitation at 488 nm and emission sampling between 505 nm and 525 nm.

### Confocal observations on fixed tissues: immunostaining

#### Ca_v_1.2 and RyR2 in isolated cardiomyocytes

Isolated CM were plated on 35 mm bottom glass Petri dishes coated with in-house prepared rat tail collagen, fixed for 1 h with 4% paraformaldehyde and permeabilised with 0.5% Triton X100 for 1 h at room temperature. Saturation with 4% BSA for 40 min was followed by double-labelling with primary antibodies against RyR2 (mouse monoclonal, Pierce) and Ca_v_1.2 (rabbit polyclonal, Calbiochem) overnight at 4 °C. Secondary antibodies (chicken anti-mouse AF488 and donkey anti-rabbit AF555, Molecular Probes) were applied for 1 h at room temperature. Immunostained cells were mounted using Mowiol 4–88 mounting medium (Sigma-Aldrich). Alexa Fluor dyes were excited at 488 and 543 nm and their fluorescence sequentially acquired with confocal microscopy (Olympus FV1000) at 515 ± 15 nm and 605 ± 50 nm, respectively. Autofluorescence and staining specificity were controlled for each preparation.

#### Cardiomyocytes in whole tissue

Freshly dissected PV were opened and fixed in 4% paraformaldehyde for 1 h at room temperature. After 7 days incubation at 4 °C with fluorescein conjugated wheat germ agglutinin (WGA, Molecular Probes), fixed tissues were mounted with Mowiol 4-88 mounting medium (Sigma-Aldrich). Image acquisition of luminal and abluminal faces was performed with confocal microscopy (Olympus FV1000) under laser excitation at 488 nm and emission sampling between 505 and 525 nm. Since PV contain several other types of cells (*e*.*g*., endothelium and vascular smooth muscle cells), cardiomyocytes were identified by their striated pattern of sarcomeres in transmitted light. This WGA labelling protocol has also been successfully used for control experiments in left ventricle and atria (Fig. S2B,C).

### Spontaneous calcium wave recording in intact cardiomyocytes

For calcium wave experiments, isolated myocytes were loaded with 5 µM Fluo-4 AM and observed through a standard fluorescence equipped inverted microscope (Nikon Diaphot 300; excitation at 480 ± 15 nm, emission at 555 ± 15 nm). They were superfused with Tyrode’s solution at 32 °C and electrically field stimulated at 1 Hz. Following the procedure of Curran *et al*.^40^, calcium fluorescence was continuously recorded, first at steady state for at least 20 s and then during the 90 s rest period. Spontaneous activity was defined as a peak signal greater than two standard deviations above the average signal for the preceding 50 ms.

### Patch-clamp experiments: SR calcium load and calcium wave-induced current

Membrane currents were recorded at room temperature (22–25 °C) with the whole cell patch clamp technique with or without simultaneous intracellular calcium measurement using K_5_-Fluo-4 fluorescence. Detailed experimental procedures are given in the supplementary data.

### Image analyses

TTorg plugin^30^ for ImageJ software was used to calculate the transverse organisation level of a TT network by performing a 2D FFT of a CM image taken at the centre of the cell and avoiding the nucleus. The amplitude of the peak in the Fourier spectrum at the TT frequency called “TT power” reflects this transverse organisation level. The detection sensibility of TT organisation (SSPD parameter) was set to 32. Tubular density on a cell slice was calculated as the ratio of the measured tubular length to the studied cell slice surface (see Fig. S6 in the supplementary data for further description).

RyR2-Ca_v_1.2 co-localisation was evaluated by two methods: image grey level profiles superposition and distance between clusters centroids^41^. For calculations of the distance separating each RyR2 and the nearest Ca_v_1.2, 2D images of longitudinal sections of cells were deconvoluted with Huygens Professional software (Scientific Volume Imaging, Brainvision). Euclidean distances between each RyR2 cluster centroid and their nearest Ca_v_1.2 cluster centroid were calculated with R software.

Calcium increase delay maps within individual CM were realised with custom routines written in ImageJ Macro language. Briefly, for each pixel in the CM, the temporal first derivative profile was computed and the temporal position of the maximum of the derivative signal was considered as the maximum rise time. A pseudo colour LUT was then applied to each pixel in function of its maximum rise time.

Sparks linescan images were analysed using SparkMaster plugin^42^ for ImageJ software. The initial parameters were 3.8 for criteria and 10 fluorescence units for background. Spark frequency, amplitude, full duration at half maximum (FDHM), full width at half maximum (FWHM), time to peak and decay time constant (tau) were studied.

### Statistics

Quantitative data are expressed as means ± SEM and the number of cells as n, except where otherwise stated. Comparisons were made using Fisher’s test (for variances) and Student’s *t*-test, Mann-Whitney rank sum test, one-way ANOVA or Kruskal-Wallis (followed by post-hoc tests) where appropriate (for means). Differences were considered significant at P < 0.05.

## Electronic supplementary material

Supplementary data

PV I shaped Calcium Transient

PV W shaped Calcium Transient

PV U shaped Calcium Transient
