# Supplementary material for: Structural heterogeneity of the rat pulmonary vein myocardium: consequences on intracellular calcium dynamics and arrhythmogenic potential
Source: Sci Rep. 2018 Feb 19;8:3244. doi: 10.1038/s41598-018-21671-9 (PMC5818479; doi:10.1038/s41598-018-21671-9)
Supplement: Supplementary file 1 — Supplementary data [file 41598_2018_21671_MOESM1_ESM.pdf]

SUPPLEMENTARY DATA

to

Structural heterogeneity of the rat pulmonary vein myocardium: consequences on intracellular calcium dynamics and arrhythmogenic potential

C. Pasqualin, A. Yu, C.O. Malécot,
F. Gannier, C. Cognard, D. Godin-Ribuot, J. Morand, P. Bredeloux, V. Maupoil

Materials and methods

1. Cardiomyocyte Isolation

Briefly, the heart and lungs were removed as one block from the anesthetised and heparinised rats and dissected in a cold (4°C) cardioplegic solution bath (in mM: 110 NaCl, 16 KCl, 1.20 $CaCl_2$, 16 $MgCl_2$, 10 $NaHCO_3$ and 10 glucose). PV and LA were chopped in small pieces and separately incubated at 37°C with 0.6 U/mL liberase™ Research grade (Roche Diagnostics) and 1 U/mL protease type XXIV (Sigma-Aldrich) in KRB solution (in mM: 35 NaCl, 4.75 KCl, 1.19 KH_2PO_4, 16 Na_2HPO_4, 25 $NaHCO_3$, 134 sucrose, 10 HEPES and 10 glucose, pH adjusted to 7.4) under 95% O_2 and 5% CO_2 until cells detachment. After enzymatic digestion, tissues were stored at room temperature in Tyrode's solution (in mM: 140 NaCl, 5.37 KCl, 1.36 $CaCl_2$, 1 $MgCl_2$, 0.33 NaH_2PO_4, 10 HEPES and 11 glucose, pH adjusted to 7.4) until use. For LV CM isolation, a similar protocol was performed using a Langendorff system with 0.1 U/mL liberase™ and 0.15 U/mL protease type XXIV in KRB solution until white areas appeared. The ventricles were then separated, cut up and stored at room temperature in Tyrode's solution until use.

2. Patch-clamp experiments and intracellular calcium measurements

Membrane currents were recorded at room temperature (22-25°C) with the whole cell patch clamp technique with or without simultaneous intracellular [Ca^{2+}] measurement using K_5-Fluo-4 fluorescence.

Dissociated cells were placed in a small chamber on the stage of an inverted microscope and continuously superfused by gravity with normal Tyrode solution. Patch pipettes were pulled from thick wall borosilicate glass capillaries (Clark Electromedical Instruments) and had resistances of 3-5 MΩ when filled with the pipette solutions. An Axopatch 200A amplifier (Axon Instruments Inc.) connected to a PC computer running Clampex (pClamp 9 software, Axon Instruments) through a Digidata 1200A interface (Axon Instruments) was used to control voltage and record currents from a holding potential (HP) of -70 mV. The data, usually acquired at 10 kHz and filtered with an 8-pole lowpass Bessel filter at 2 kHz, were analysed offline with Clampfit. Membrane capacitance (C_m) was measured by integration of the capacitive currents in response to a series of 10 hyperpolarising pulses applied from the HP (amplitude and duration: 10 mV, 10 ms) and then averaged. The pipette and cell capacitances were compensated by 80 %.

2.1. SR Ca^{2+} load determination

Steady-state SR Ca^{2+} load was induced by applying a 100 ms voltage step from -70 mV to -40 mV (to inactivate the fast Na current) followed by a 300 ms voltage step from -40 to +10 mV (to maximally activate the calcium current) at 1 Hz for at least 2 min to allow SR calcium full replenishment (almost 90 s are necessary for the calcium transient to reach its steady-state amplitude after caffeine application in PV CM - not shown). Thus, this protocol avoids underestimation of the SR content. Upon cessation of stimulation with the cell voltage-clamped at -70 mV, the extracellular solution directly superfused onto the cell (fast in-house electro valve-controlled perfusion system) was switched to one containing 10 mM caffeine to induce SR Ca^{2+} release. The integral of the inward current generated in response to caffeine (sodium-calcium exchange current, I_{NCX}) was used to calculate the SR Ca^{2+} content (Table S1). No correction was made for non-sodium-calcium exchange-mediated calcium efflux. During these experiments, the extracellular solution contained (in mM): 130 NaCl, 10 CsCl, 2 $CaCl_2$, 1 $MgCl_2$, 5 4-aminopyridine, 10 HEPES buffer, 10 glucose, pH 7.4 with NaOH. Patch pipettes were filled with a solution based on Macquaide *et al*. (2015) and containing (in mM): 120 aspartic acid, 10 TEACl, 5 ATP-Mg, 0.5 $MgCl_2$, 10 HEPES buffer, pH 7.2 with CsOH.

2.2. Spontaneous calcium wave-induced current

Considering the low calcium wave frequency observed in intact isolated cardiomyocytes, waves were induced by applying a 100 ms voltage ramp from -70 mV to -40 mV (to inactivate the fast Na current) followed by a 300 ms voltage step from -40 to +10 mV (to maximally activate the calcium current) at 0.5 Hz for at least 2 min to allow SR calcium full replenishment. Calcium waves and corresponding inward current were immediately and simultaneously recorded during rest at -70 mV, a potential close to the resting potential. Since rat PV is less polarised at rest than LA tissue [1,2], current recording was preferred over DADs recording in current clamp mode for direct comparison between PV and LA in the same experimental conditions. Fluo-4 was excited between 485 and 495 nm with an Olympus mercury lamp and fluorescence collected between 515 and 555 nm via a photomultiplier connected to a Dual mode Photo-detection System Cairn Integra (Cairn Research Ltd, UK) used in average mode (average bandwidth: 200 or 500 Hz). Calcium wave amplitudes are expressed as F/F_0. During these experiments, the extracellular solution contained (in mM): 130 NaCl, 10 CsCl, 1.36 $CaCl_2$, 1 $MgCl_2$, 5 4-aminopyridine, 10 HEPES buffer, 10 glucose, pH 7.4 with NaOH. Patch pipettes were filled with a solution based on Voigt *et al*. (2012) and containing (in mM): 92 K-Aspartate, 48 KCl, 1 ATP-Mg, 4 ATP-Na_2, 0.1 GTP-Tris, 0.02 EGTA, 10 HEPES buffer, 0.05 K_5-Fluo-4, pH 7.2 with KOH.

Table S1. Caffeine-induced NCX current and SR Ca load in LA and PV cardiomyocytes at 25°C.

	LA CM (n)	PV CM (n)	
C_m (pF)	54.60 ± 3.92 (20) *	92.42 ± 4.96 (24) *	
C_m/V ratio[(1)] (pF/pL)	14.6	14.6 (LA value)	6.76 (LV value)
Cell volume[(2)] (pL)	3.76 ± 0.32 (15) ‡	6.19 ± 0.37 (21) ≠‡	13.37 ± 0.80 (21) ≠‡
**Caffeine-induced NCX current at -70 mV**[(3)]			
Peak amplitude (pA)	-148.07 ± 13.07 (20)	-190.55 ± 13.71 (24)	
Peak amplitude (pA/pF)	-2.79 ± 0.18 (20) *	-2.03 ± 0.13 (24) *	
Time to Peak (ms)	100.95 ± 6.97 (20)	110.22 ± 9.36 (24)	
Half width (ms)	464.44 ± 78.89 (19)	480.56 ± 32.52 (24)	
10-90% decay (ms)	2516.54 ± 350.81 (19)	1813.37 ± 150.69 (24)	
**SR Ca load**[(4)]			
Ca^{++} load (fmol)	2.23 ± 0.19 (15)	2.32 ± 0.16 (21)	
Ca^{++} load (attomol/pF)	41.18 ± 3.39 (15) *	26.15 ± 1.56 (21) *	
[Ca^{++}] (µM)[(5)]	601.21 ± 49.47 (15) ‡	381.87 ± 22.74 (21) ≠‡	176.81 ± 10.53 (21) ≠‡

C_m: cardiomyocytes membrane capacitance in this study.
(1): C_m/V: capacitance/cell volume ratio. Values for LA and LV according to Walden *et al.* (2009) and Satoh *et al.* (1996), respectively.
(2): calculated from above C_m values and C_m/V ratios.
(3): following 2 min stimulation at 1 Hz (2 mM external Ca^{++}, 300 ms pulses from -40 to +10 mV) – 10 mM caffeine.
(4): calculated from the caffeine-induced NCX current.
(5): calculated with the capacitance/cell volume ratio and expressed relative to the CM volume.
Data are expressed as mean values ± SE. The number of cardiomyocytes from which data could be effectively measured is indicated in parenthesis.
*****, **‡**: P<0.05, PV *vs*. LA; **≠**: PV *vs.* PV. ANOVA on ranks and one-way ANOVA followed by Dunn's or Holm-Sidak's post-hoc tests or Rank sum or Student's *t*-test where appropriate.

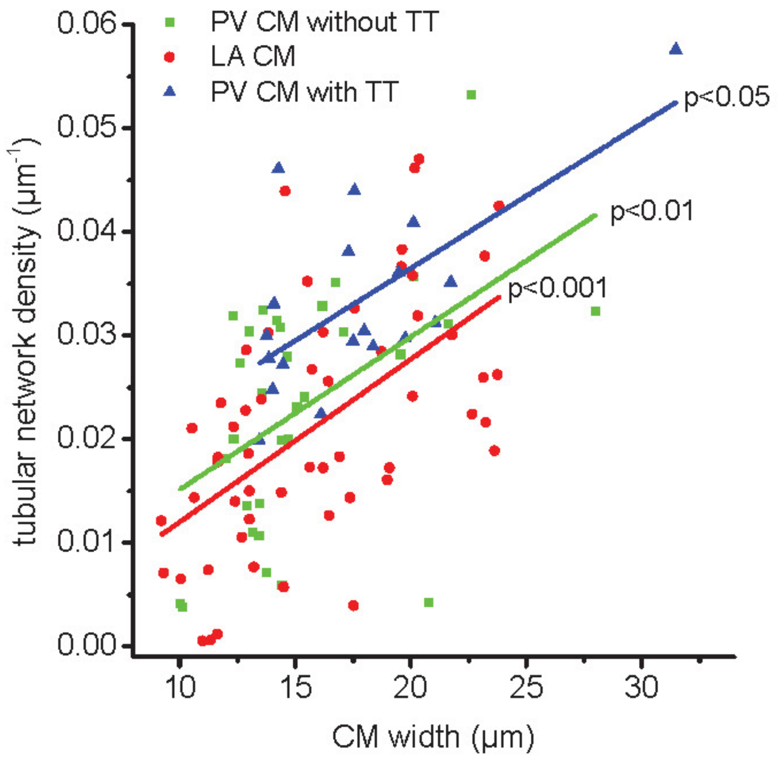

Figure S1. Relation between the tubular network length/area ratio and the cardiomyocyte width in PV CM with TT (blue), without TT (green) and in LA CM (red) cardiomyocytes. For each type of cardiomyocyte, correlation was tested with Pearson's correlation test.

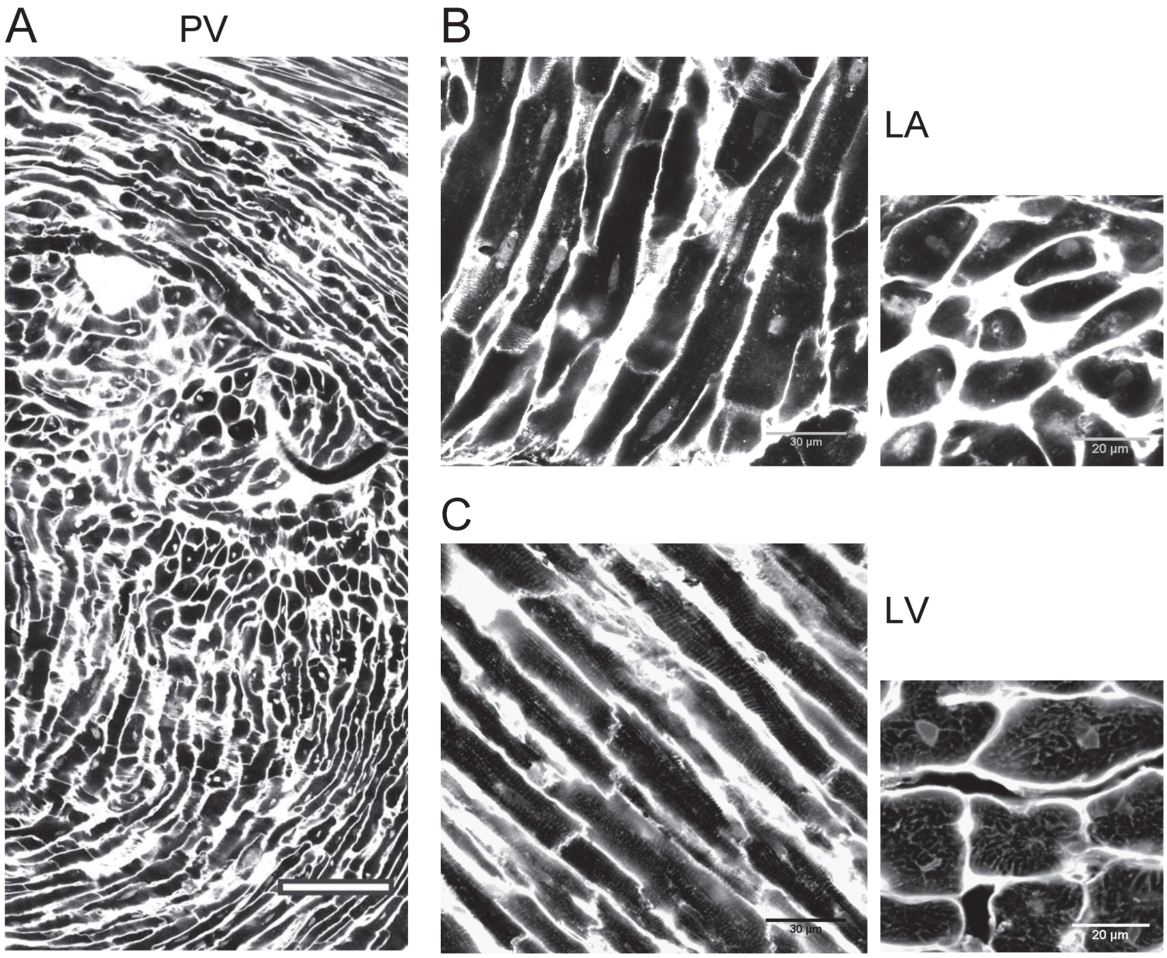

Figure S2. Fluorescent WGA labelling of CM in PV, LA and LV tissues and observation in confocal microscopy. A, composite of circular and orthogonal orientations in PV CM layer (luminal face). Scale bar represents 100 µm. B and C, representative panels of CM in LA (B) and LV (C) tissues, on longitudinal (left) and transversal (right) sections.

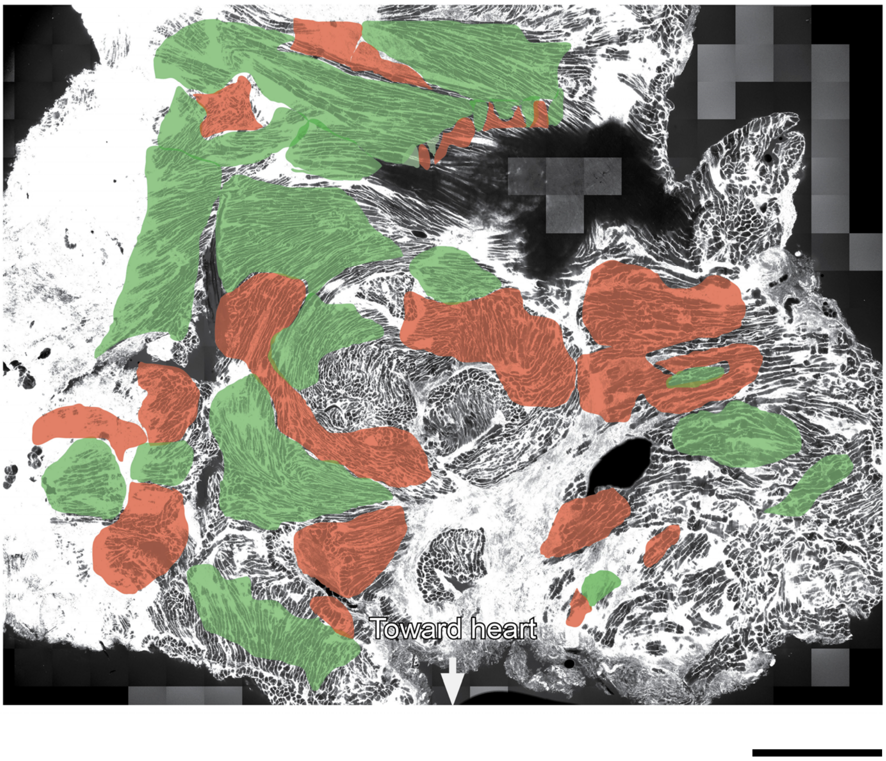

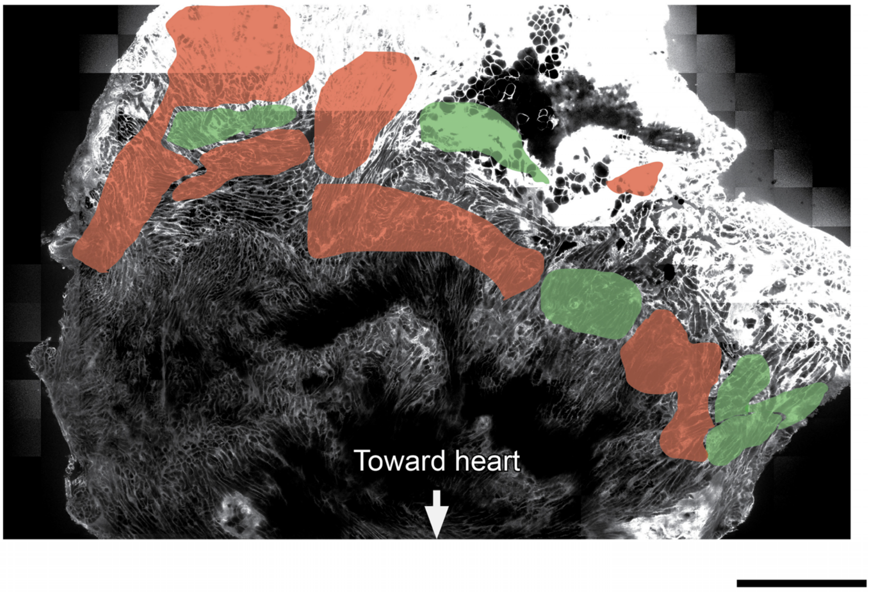

Figure. S3. Two examples of the distribution of groups of different types of CM in whole mounts of right superior PV. The whole mounts were stained with wheat germ agglutinin and observed under confocal microscopy at sufficient magnification (0.26 µm/pixel) to evaluate tubule systems in individual CM. These figures represent mosaics made up of ~ 500 individual images which have been overlaid with a mask which obscures the detail shown in Fig. 2 but which indicates regions of the PV luminal surface showing CM with transversely organized tubules (Green) and CM without transversely organized tubules (Red). Scale bars represent 200 µm.

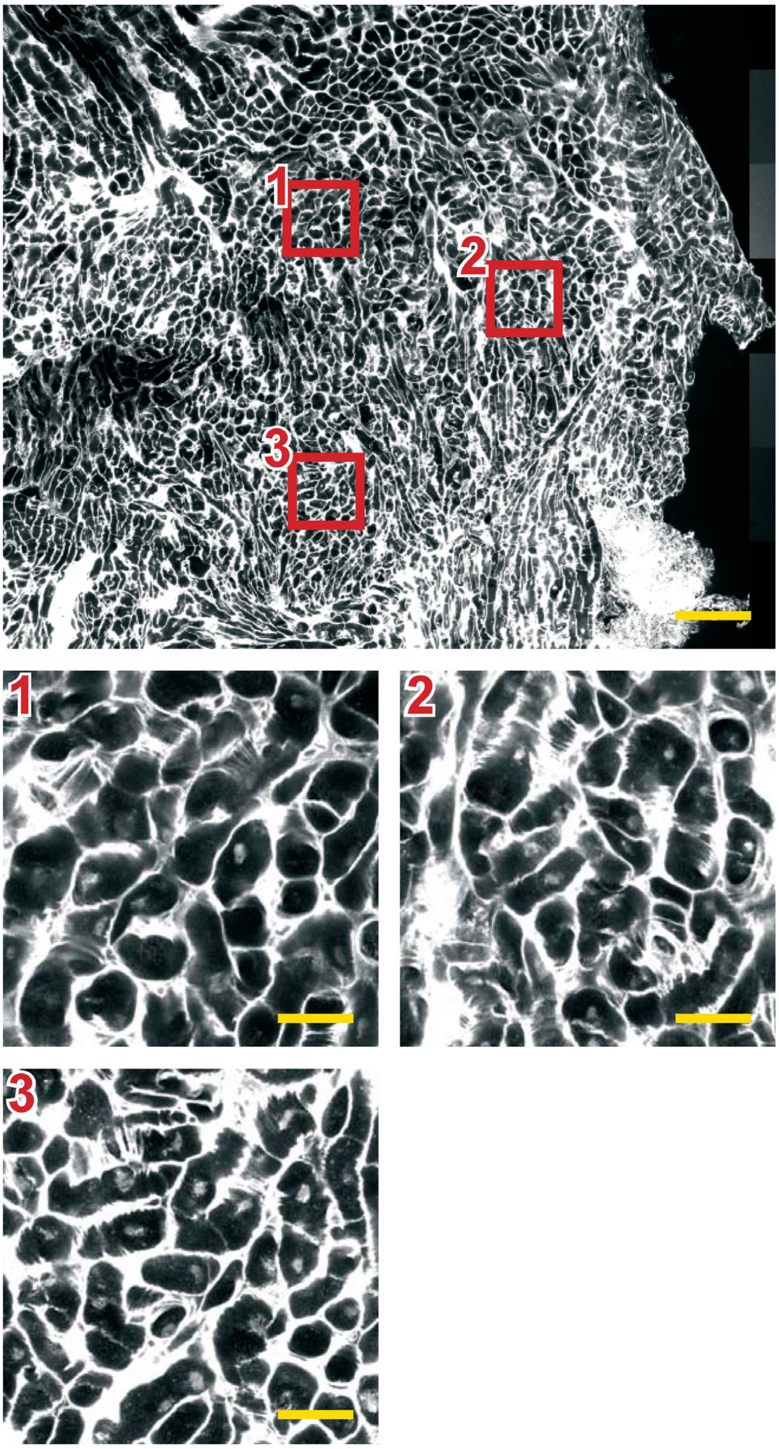

Figure S4. Representative example of a left atria slice stained with WGA and observed in confocal microscopy. Panels 1 to 3 correspond to a magnification of the areas outlined by the red squares shown in the top panel. Few or no tubules were observed throughout the atrial slices (n=4 rats). Scale bars represent 100 µm in the top panel and 20 µm otherwise.

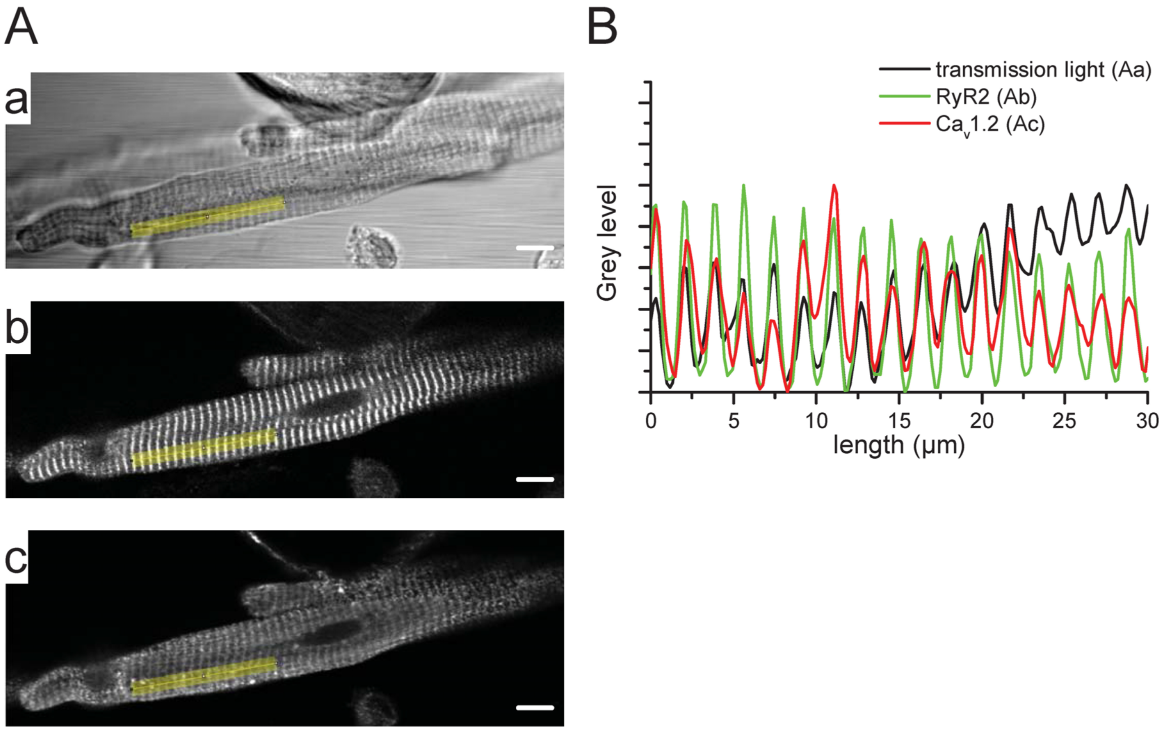

Figure S5. A, PV cardiomyocyte in transmitted light microscopy (a) and in confocal microscopy with labeled RyR2 (b) and Cav1.2 (c). Scale bars represent 10 µm. B, grey level profiles along the yellow line drawn in A images.

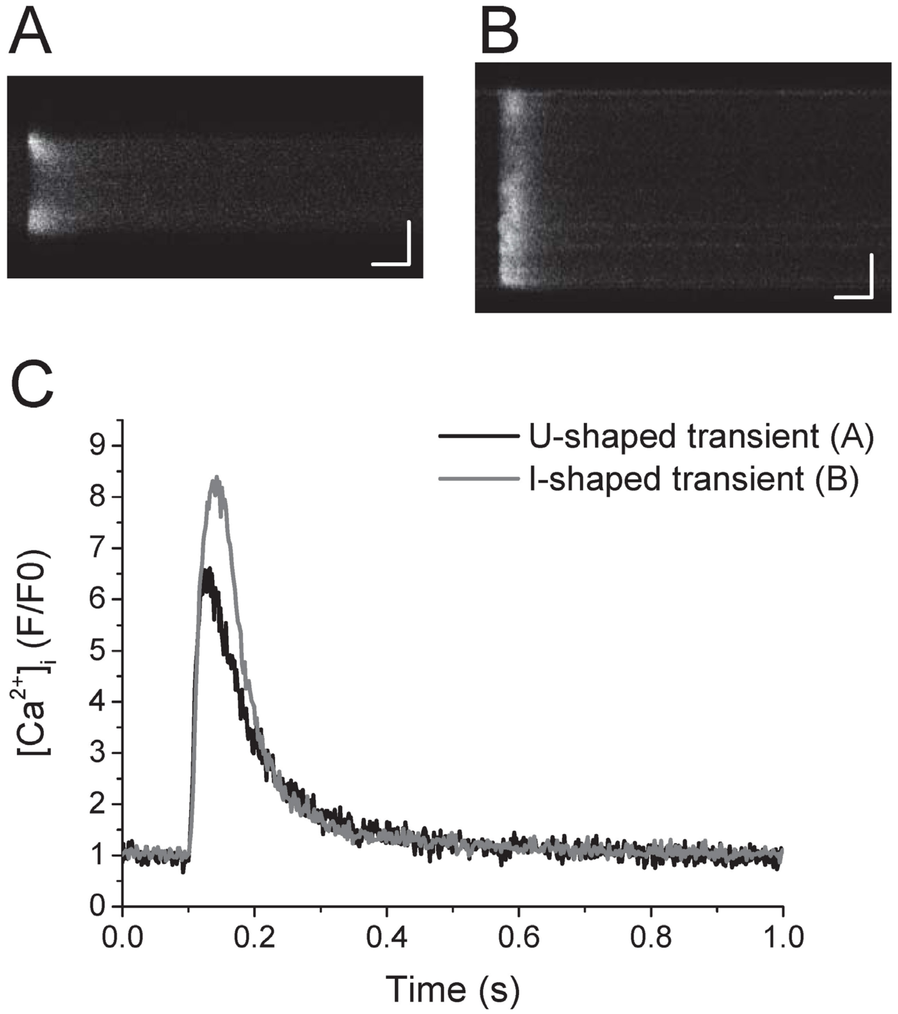

Figure S6. Comparison of the amplitudes of the cytoplasmic calcium rise for *U*- and *I*-shaped electrically stimulated calcium transients in PV cardiomyocytes. A and B, examples of *U*- and *I*-shaped calcium transients recorded along a line drawn transversally across two different Fluo-4 loaded PV CM and recorded in confocal microscopy. Vertical and horizontal scale bars represent respectively 5 µm and 100 ms. C, Y axis projection of A and B calcium transients realized with SarConfoCal plugin[3] for ImageJ software.

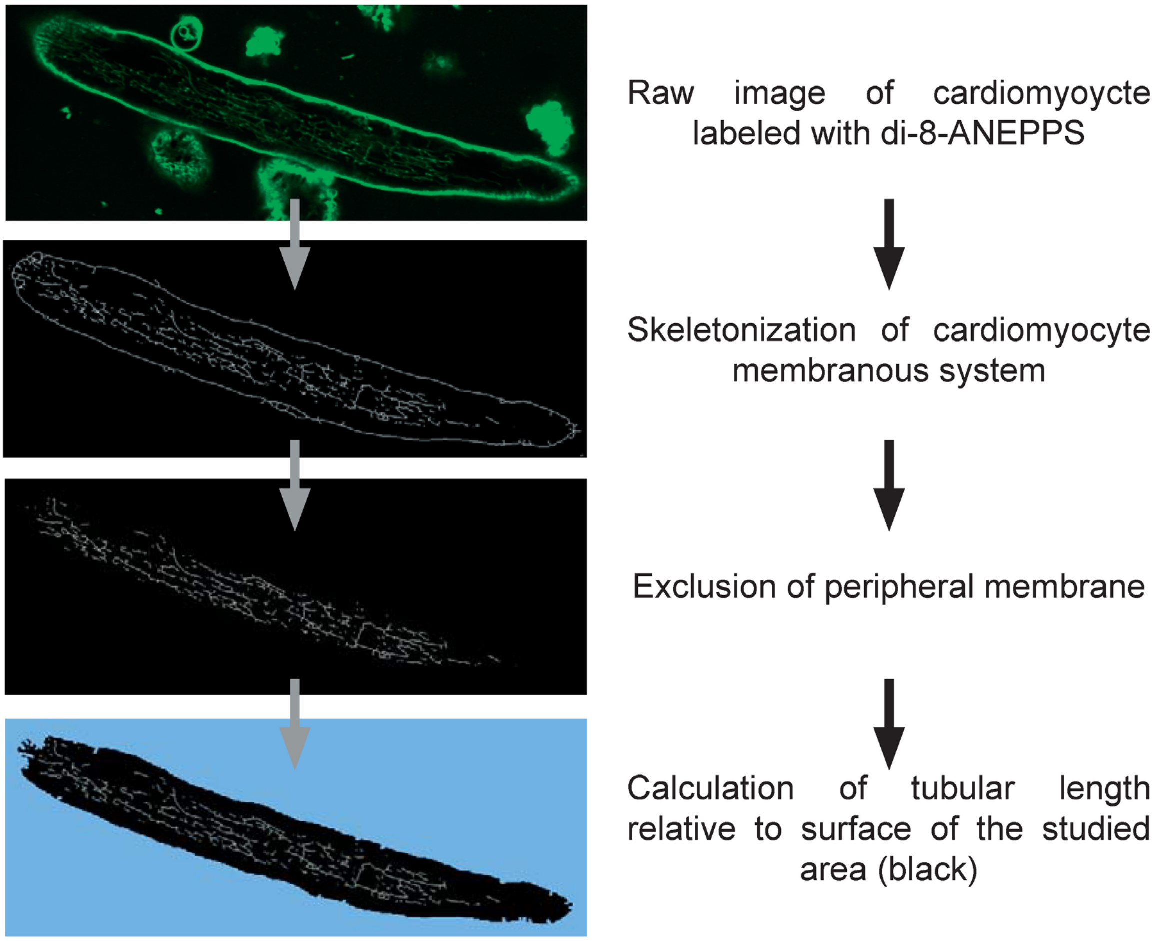

Figure S7. Analysis workflow for the determination of tubular density in cardiomyocytes. All processing was done with ImageJ custom routines.

References

1. Doisne, N., Maupoil, V., Cosnay, P. & Findlay, I. Catecholaminergic automatic activity in the rat pulmonary vein: electrophysiological differences between cardiac muscle in the left atrium and pulmonary vein. *Am. J. Physiol. Heart Circ. Physiol.* **297,** H102-108 (2009).
2. Malécot, C. O., Bredeloux, P., Findlay, I. & Maupoil, V. A TTX-Sensitive Resting Na+ Permeability Contributes to the Catecholaminergic Automatic Activity in Rat Pulmonary Vein. *J. Cardiovasc. Electrophysiol.* **26,** 311–319 (2015).
3. Pasqualin, C. *et al.* SarConfoCal: simultaneous sarcomere length and cytoplasmic calcium measurements for laser scanning confocal microscopy images. *Bioinformatics* **33,** 789–790 (2017).
4. Macquaide, N., Bito, V. & Sipido, K. R. Measuring sarcoplasmic reticulum Ca2+ content, fractional release, and Ca2+ buffering in cardiac myocytes. *Cold Spring Harb. Protoc.* **2015,** 403–407 (2015).
5. Voigt, N. *et al.* Enhanced Sarcoplasmic Reticulum Ca2+ Leak and Increased Na+-Ca2+ Exchanger Function Underlie Delayed Afterdepolarizations in Patients With Chronic Atrial Fibrillation. *Circulation* **125,** 2059–2070 (2012).
